# Assessment of residual body weight gain and residual intake and body weight gain as feed efficiency traits in the turkey *(Meleagris gallopavo)*

**DOI:** 10.1186/1297-9686-45-26

**Published:** 2013-07-16

**Authors:** Owen W Willems, Stephen P Miller, Benjamin J Wood

**Affiliations:** 1 Centre for the Genetic Improvement of Livestock, Department of Animal and Poultry Science, University of Guelph, Guelph, ON, Canada; 2 Hybrid Turkeys, Suite C, 650 Riverbend Drive, Kitchener, ON, Canada

## Abstract

**Background:**

Since feed represents 70% of the total cost in poultry production systems, an animal’s ability to convert feed is an important trait. In this study, residual feed intake (RFI) and residual body weight gain (RG), and their linear combination into residual feed intake and body weight gain (RIG) were studied to estimate their genetic parameters and analyze the potential differences in feed intake between the top ranked birds based on the criteria for each trait.

**Methods:**

Phenotypic and genetic analyses were completed on 8340 growing tom turkeys that were measured for feed intake and body weight gain over a four-week period from 16 to 20 weeks of age.

**Results:**

The heritabilities of RG and RIG were 0.19 ± 0.03 and 0.23 ± 0.03, respectively. Residual body weight gain had moderate genetic correlations with feed intake (−0.41) and body weight gain (0.43). All three linear combinations to form the RIG traits had genetic correlations ranging from −0.62 to −0.52 with feed intake, and slightly weaker, 0.22 to 0.34, with body weight gain. Sorted into three equal groups (low, medium, high) based on RG, the most efficient group (high) gained 0.62 and 1.70 kg more (*P* < 0.001) body weight than that of the medium and low groups, yet the feed intake for the high group was less (*P* < 0.05) than that of the medium group (19.52 vs. 19.75 kg). When separated into similar partitions, the high RIG group (most efficient) had both the lowest (*P* < 0.001) feed intake (18.86 vs. 19.57 and 20.41 kg) and the highest (*P* < 0.001) body weight gain (7.41 vs. 7.03 and 6.43 kg) relative to the medium and low groups, respectively.

**Conclusions:**

The difference in feed intake between the top ranked birds based on different residual feed efficiency traits may be small when looking at the average individual, however, when extrapolated to the production level, the lower feed intake values could lead to significant savings in feed costs over time.

## Background

Feed efficiency is important to animal production because feed cost is a large component of the overall cost in all production settings. A number of measures have been used to define feed efficiency at the herd, flock or individual level and each measure has its own advantages and disadvantages. The most commonly used measures are feed conversion ratio (FCR) and residual feed intake (RFI) [1]. Feed conversion ratio is defined as the amount of feed consumed per unit of weight gain, and is a composite trait of start and end body weights and feed intake. Selection for FCR can lead to unexpected consequences because of the relationship between the two traits that make up FCR. When selection intensity increases, direct selection on FCR focuses selection primarily on the information in the numerator, regardless of the distributional properties of the components [2]. An additional problem is that ratio traits, such as FCR, cannot accommodate differential economic weights between the two traits involved [3].

Residual feed intake (RFI) was originally used in beef cattle by Koch et al. [4] and in poultry by Luiting [5]. Residual feed intake represents the amount of feed intake which is not accounted for by the expected requirements for production (e.g. milk and egg production or in this case body weight gain) and body weight maintenance [6]. Some of the cited advantages of RFI are that it is heritable, moderately correlated with FCR and feed intake, and ideally, independent of production traits [6,7]. Although RFI may be seen as a good indicator of feed efficiency, it does have its drawbacks, namely, slow growing animals eating a relatively small amount of feed may have a more favorable RFI value [8]. Previously reported heritability estimates for FCR and RFI in the turkey were 0.22 and 0.24, respectively, with a genetic correlation of 0.80 [7].

Similar to RFI, residual body weight gain (RG) is defined as the difference between actual and predicted body weight gain based on the regression of body weight gain on metabolic mid-weight (MMW) and feed intake [9]. Hence, RG represents the amount of body weight gain not accounted for by differences in feed intake and MMW. In general, RG is associated with faster growth rates while remaining independent of differences in feed intake. In contrast to RFI (where a negative value is beneficial), a positive value for RG is favorable.

Residual intake and body weight gain (RIG), developed in beef cattle by Berry and Crowley [8], is a linear combination of RFI and RG. Because it is a linear combination, it is hypothesized that RIG offers the benefits of both components of feed efficiency. Currently, no genetic parameter estimates for RG or RIG for turkeys are reported in the literature. The aim of the current study was to estimate the genetic and phenotypic parameters for both RG and RIG in a commercial turkey breeding population.

## Methods

The industry source Hybrid Turkeys collected all data utilized in this analysis. Hybrid Turkeys has an animal welfare committee that ensures the welfare of the turkeys meets the appropriate standards of care.

### Turkey population

Toms from a male primary breeder turkey line (n = 8340) with data collected over a 10-year period with pedigree information (28 464 relatives of the birds with records) were used. Rearing until 15 weeks of age was under a standard commercial production environment and feeding regime, which involved group housing with shared feeders and drinkers. At 15 weeks of age, toms were placed in individual cages (0.60 m wide, 0.85 m long and 0.82 m high) to acclimatize and they remained in the same cage throughout the feeding trial, from approximately 16 to 20 weeks of age. Body weight was measured at the start (16 weeks of age) and the end of the trial (20 weeks of age). Feed intake was recorded by weighing feeders at the beginning of the trial, weighing the feed added to each feeder and weighing the remaining feed at the end of the trial. During this period, toms were fed a standard commercial diet, as shown in Table 1. Feed was available *ad libitum,* with access to feed from individual feeders within a cage and a shared water source between cages.

**Table 1:** **Commercial diet fed*ad libitum*to growing tom turkeys for the four-week test period from 16 to 20 weeks of age**

**Nutrient composition**	**Units**	**Amount**
Crude protein	%	27.5
ME	MJ/kg	11.92
ME	kcal/lb	1292
ME	kcal/kg	2850
Available lysine	%	1.62
Available arginine	%	1.64
Available methionine	%	0.65
Available methionine + cysteine	%	1.05
Available threonine	%	0.96
Available tryptophan	%	0.27
Available valine	%	1.13
Available Isoleucine	%	1.00
Calcium total	%	1.40
Available phosphorus	%	0.75
Sodium total	%	0.17

### Data analysis

Average daily gain (*ADG*) was calculated as:

ADG=weightatendoftrialkg−weightatstartoftrialkgdaysontrial

Daily feed intake (*DFI*) was calculated as:

DFI=totalfeedconsumedkgdaysontrial

Feed conversion ratio (*FCR*) was calculated as:

FCR=feedconsumedkgbodyweightgainkg

Metabolic mid-weight (*MMW*) was calculated as:

MMW=weightatstartoftrialkg+weightatendoftrialkg20.75

To determine RFI, expected feed intake was calculated as a multiple regression with observed feed intake as the dependent variable (Model 1). To determine RG, expected body weight gain was calculated as a multiple regression with observed body weight gain as the dependent variable (Model 2).

Model 1: FI=μ+b1MMW+b2WG+hatch+e

Model 2: *WG* = *μ* + *b*_3_*MMW* + *b*_4_*FI* + *hatch* + *e*

In Models 1 and 2, *FI* is feed intake over the test period, *MMW* is the metabolic mid-weight, *WG* is body weight gain over the test period, *μ* is the intercept, *b*_*1*_, *b*_*2*_, *b*_*3*_, and *b*_*4*_ are partial regression coefficients, and *e* is the residual. The fixed contemporary group effect (*hatch*) was used to account for the common environment effect that influenced birds hatched on the same date and managed in the same group. Regression coefficients from Models 1 and 2 were used to calculate RFI (Model 3) and RG (Model 4) respectively:

Model 3: RFI=FI−μ+b^1MMW+b^2WG

Model 4: RG=WG−μ+b^3MMW+b^4FI

Residual intake and body weight gain (RIG) was calculated as RG-RFI, after standardizing both RG and RFI to a variance of 1. Taking the negative of RFI puts both RFI and RG on a positive scale, allowing for their combination into the RIG value. Residual intake and body weight gain is a linear function of both RFI and RG, which are themselves, linear functions of their component traits: feed intake, body weight gain and starting body weight. Two other combinations of RFI and RG were assessed to analyze what may be closer to an optimized emphasis. Residual intake and body weight gain 2 (RIG2) was computed as 2RG-RFI, residual intake and body weight gain 3 (RIG3) as RG-2RFI.

The 8340 toms were split evenly into three groups: high, medium and low, separately for RFI, RG and RIG. Means of the feed efficiency and performance traits from the three groups were tested with Tukey multiple comparisons tests using the GLM procedure in SAS. For the total feed intake analysis, the 8340 toms were separately ranked based on RFI, RG, RIG, RIG2 and RIG3. From these data, the top 5% and 1% of birds, as well as the bottom 5% for each trait were chosen and a phenotypic analysis of DFI, body weight gain, and ADG was performed. Means were calculated for the sorted data for DFI, body weight gain and average daily gain. The number of days to achieve a 5 kg gain in body weight was calculated as 5 kg divided by the ADG for each scenario. Total feed intake over the duration was calculated by multiplying the DFI by the number of days to achieve a body weight gain of 5 kg.

### Genetic analyses

Heritability for each trait together with phenotypic and genetic correlations were estimated using ASREML 2.0 [10]. The model for all traits was:

Trait=hatch+animal+e,

where *trait* is RFI, RG, RIG, RIG2, RIG3, FCR, feed intake, MMW, or body weight gain. *Hatch* was a fixed contemporary group effect to account for the common environment of group of birds hatched and managed together, *animal* represents the random additive genetic effect, and *e* the residual random effect. The random effects were assumed to be normally distributed with a mean of 0. Heritability estimates were calculated based on a single trait model. Phenotypic and genetic correlations were estimated pair-wise using a bivariate model.

## Results

Phenotypic means, standard deviations and heritabilities of each trait are in Table 2. The means for RFI, RG, RIG, RIG2 and RIG3 were equal to 0, since they represent residuals of a linear model. All traits had a moderate heritability between 0.13 and 0.30, with the exception of FCR, which had a heritability of 0.05. Phenotypic and genetic correlations are in Table 3. The phenotypic correlations between feed intake, MMW and body weight gain were positive, ranging from 0.41 to 0.73, while the genetic correlations ranged from 0.62 to 0.67. Feed conversion ratio was phenotypically negatively correlated with feed intake (−0.27) but had a positive genetic correlation (0.21). Feed conversion ratio was also negatively correlated with body weight gain, both phenotypically (−0.68) and genetically (−0.64).

**Table 2:** Mean performance, phenotypic standard deviation (SD) and heritability (standard error) for the 8340 tom turkeys over the four-week test period

**Trait**^**1**^	**Mean**	**SD**	**Heritability**
FI (kg)	19.61	3.16	0.20 (0.03)
MMW (kg)	1.49	0.11	0.30 (0.03)
BW16 (kg)	13.62	1.51	0.30 (0.03)
BW20 (kg)	20.58	2.12	0.23 (0.03)
WG (kg)	6.96	1.52	0.13 (0.02)
FCR (kg/kg)	2.95	0.96	0.05 (0.02)
RFI (kg)	0	1.46	0.23 (0.03)
RG (kg)	0	0.87	0.19 (0.03)
RIG	0	1.89	0.23 (0.03)
RIG2	0	1.43	0.22 (0.03)
RIG3	0	1.43	0.24 (0.03)

^1^Feed intake (FI), metabolic mid-weight (MMW), 16-week body weight (BW16), 20-week body weight (BW20), body weight gain (WG), feed conversion ratio (FCR), residual body weight gain (RG), residual feed intake (RFI), residual intake and body weight gain (RIG, RIG2 and RIG3).

**Table 3:** **Phenotypic and genetic correlations**^**2**^**, above and below the diagonal, respectively, with standard errors (in brackets) for the 8340 tom turkeys**

**Traits**^**1**^	**FI**	**MMW**	**WG**	**FCR**	**RFI**	**RG**	**RIG**	**RIG2**	**RIG3**
FI		0.41 (0.01)	0.73 (0.01)	−0.27 (0.01)	0.61 (0.01)	0.00 (0.01)	−0.31 (0.01)	−0.20 (0.01)	−0.42 (0.01)
MMW	0.67 (0.06)		0.59 (0.01)	0.05 (0.01)	0.00 (0.01)	0.00 (0.01)	0.00 (0.01)	0.00 (0.01)	0.00 (0.01)
WG	0.63 (0.05)	0.62 (0.06)		−0.68 (0.01)	0.00 (0.01)	0.66 (0.01)	0.42 (0.01)	0.52 (0.01)	0.29 (0.01)
FCR	0.21 (0.16)	−0.04 (0.15)	−0.64 (0.11)		0.15 (0.01)	−0.71 (0.01)	−0.59 (0.01)	−0.64 (0.00)	−0.51 (0.01)
RFI	0.67 (0.06)	0.09 (0.09)	−0.07 (0.11)	0.36 (0.09)		−0.58 (0.01)	−0.86 (0.00)	−0.77 (0.01)	-
RG	−0.41 (0.09)	−0.28 (0.09)	0.43 (0.08)	−0.66 (0.10)	−0.76 (0.05)		0.91 (0.00)	-	-
RIG	−0.57 (0.07)	−0.22 (0.09)	0.29 (0.10)	−0.93 (0.09)	−0.93 (0.02)	0.94 (0.01)		-	-
RIG2	−0.52 (0.08)	−0.25 (0.09)	0.34 (0.09)	−0.94 (0.12)	−0.87 (0.03)	-	-		-
RIG3	−0.62 (0.06)	−0.18 (0.09)	0.22 (0.10)	−0.89 (0.10)	-	-	-	-	

^1^Feed intake (FI), metabolic mid-weight (MMW), body weight gain (WG), feed conversion ratio (FCR), residual feed intake (RFI), residual body weight gain (RG), residual intake and body weight gain (RIG, RIG2 and RIG3); ^2^due to confounding factors from linear combinations of RFI and RG into RIG traits, correlations were not calculated in some instances.

None of the residual feed efficiency traits (RG, RFI, RIG, RIG2, RIG3) were phenotypically correlated with MMW, as expected, however weak genetic correlations did exist (−0.28 to 0.09). Residual body weight gain was moderately correlated, phenotypically and genetically, with body weight gain (0.66 and 0.43), FCR (−0.71 and −0.66), strongly correlated with RIG (0.91 and 0.94), and had a zero phenotypic and a moderate negative (−0.41) genetic correlation with feed intake. Residual feed intake showed a zero phenotypic correlation with weight gain, along with a weak negative genetic correlation (−0.07). Residual feed intake also had a weak positive genetic correlation with FCR (0.36), along with a strong negative genetic correlation with RG (−0.76). Residual intake and body weight gain was phenotypically and genetically negatively correlated with feed intake (−0.31 and −0.57) and FCR (−0.59 and −0.93) but was positively correlated with body weight gain (0.42 and 0.29, respectively). For RIG2, phenotypic and genetic correlations of −0.20 and −0.52 were seen with feed intake, and of 0.52 and 0.34 with body weight gain. For RIG3, phenotypic correlations were −0.42 and 0.29 with feed intake and body weight gain, respectively.

The 8340 turkey toms were separated into three equally sized groups based on their ranking for RFI, RG and RIG, as shown in Tables 4, 5 and 6, respectively. The most efficient RFI group (low) had lower feed intake values (*P* < 0.001) than the medium and high groups (18.16 vs. 19.51 and 21.16 kg) (Table 4). However, the low RFI group also had the lowest (*P* < 0.001) body weight gain (6.86 vs. 7.01 and 7.00 kg). Additionally, the low RFI group was superior to the medium and high groups (*P* < 0.001) for FCR, RG and all three RIG traits. There was no difference (*P* > 0.05) between the three groups for 16 and 20-week body weights or MMW.

**Table 4:** Least square means for feed efficiency and growth traits for 8340 tom turkeys of groups ranked high, medium and low for residual feed intake and Tukey multiple comparisons within a row

**Trait**	**Residual feed intake group**^**1**^			**P-value**^**2**^
	**Low**	**Medium**	**High**	
Residual feed intake, kg	−1.38^a^	−0.10^b^	1.48^c^	< 0.001
Body weight 16 weeks, kg	13.65	13.60	13.61	0.50
Body weight 20 weeks, kg	20.51	20.61	20.62	0.11
Feed intake, kg	18.16^a^	19.51^b^	21.16^c^	< 0.001
Body weight gain, kg	6.86^a^	7.01^b^	7.00^b^	< 0.001
Metabolic mid-weight, kg	1.49	1.49	1.50	0.66
Feed conversion ratio	2.78^a^	2.88^b^	3.20^c^	< 0.001
Residual body weight gain, kg	0.43^a^	0.06^b^	−0.49^c^	< 0.001
Residual intake and body weight gain	1.44^a^	0.14^b^	−1.57^c^	< 0.001
Residual intake and body weight gain 2	0.96^a^	0.10^b^	−1.07^c^	< 0.001
Residual intake and body weight gain 3	1.19^a^	0.10^b^	−1.30^c^	< 0.001

^a,b,c^Within a row, means with different superscript letters differ for multiple comparisons according to Tukey test (*P* < 0.05); ^1^residual feed intake groups created by dividing the dataset equally into three groups based on residual feed intake; ^2^significance of the group effect.

**Table 5:** Least square means for feed efficiency and growth traits for 8340 tom turkeys of groups ranked high, medium and low for residual body weight gain and Tukey multiple comparisons within a row

**Trait**	**Residual body weight gain group**^**1**^			**P-value**^**2**^
	**High**	**Medium**	**Low**	
Residual body weight gain, kg	0.85^a^	0.06^b^	−0.91^c^	< 0.001
Body weight 16 weeks, kg	13.10^a^	13.67^b^	14.09^c^	< 0.001
Body weight 20 weeks, kg	20.84^a^	20.78^a^	20.12^b^	< 0.001
Feed intake, kg	19.52^a^	19.75^b^	19.56^ab^	0.01
Body weight gain, kg	7.73^a^	7.11^b^	6.03^c^	< 0.001
Metabolic mid-weight, kg	1.49	1.50	1.49	0.19
Feed conversion ratio	2.54^a^	2.80^b^	3.52^c^	< 0.001
Residual feed intake, kg	−0.77^a^	−0.07^b^	0.83^c^	< 0.001
Residual intake and body weight gain	1.50^a^	0.11^b^	−1.61^c^	< 0.001
Residual intake and body weight gain 2	1.24^a^	0.09^b^	−1.32^c^	< 0.001
Residual intake and body weight gain 3	1.01^a^	0.08^b^	−1.09^c^	< 0.001

^a,b,c^Within a row, means with different superscript letters differ for multiple comparisons according to Tukey test (*P* < 0.05); ^1^residual body weight gain groups created by dividing the dataset equally into three groups based on residual body weight gain; ^2^significance of the group effect.

**Table 6:** Least square means for feed efficiency and growth traits for 8340 tom turkeys of groups ranked high, medium and low for residual intake and body weight gain and Tukey multiple comparisons within a row

**Trait**	**Residual intake and body weight gain group**^**1**^			**P-value**^**2**^
	**High**	**Medium**	**Low**	
Residual intake and body weight gain	1.68^a^	0.20^b^	−1.81^c^	< 0.001
Body weight 16 weeks, kg	13.34^a^	13.63^b^	13.90^c^	< 0.001
Body weight 20 weeks, kg	20.75^a^	20.66^a^	20.33^b^	< 0.001
Feed intake, kg	18.86^a^	19.57^b^	20.41^c^	< 0.001
Body weight gain, kg	7.41^a^	7.03^b^	6.43^c^	< 0.001
Metabolic mid-weight, kg	1.49	1.50	1.49	0.06
Feed conversion ratio	2.57^a^	2.84^b^	3.44^c^	< 0.001
Residual feed intake, kg	−1.20^a^	−0.01^b^	1.29^c^	< 0.001
Residual body weight gain, kg	0.75^a^	0.05^b^	−0.80^c^	< 0.001
Residual intake and body weight gain 2	1.27^a^	0.09^b^	−1.36^c^	< 0.001
Residual intake and body weight gain 3	1.25^a^	0.10^b^	−1.35^c^	< 0.001

^a,b,c^Within a row, means with different superscript letters differ for multiple comparisons according to Tukey test (*P* < 0.05); ^1^Residual intake and body weight gain groups created by dividing the dataset equally into three groups based on residual intake and body weight gain; ^2^Significance of the group effect.

The results of the RG toms separated into groups are in Table 5. The high RG group (most efficient) gained 0.62 and 1.70 kg more (*P* < 0.001) body weight than the medium and low groups. The feed intake was lower (*P* < 0.05) for the high group than for the medium group (19.52 vs. 19.75 kg) but was similar (*P* > 0.05) to the low group (19.56 kg). Sixteen-week body weight was lowest (*P* < 0.001) in the high group compared to the medium and low groups (13.10 vs. 13.67 vs. 14.09 kg). The high RG group was also significantly better (*P* < 0.001) for FCR, RFI and all three RIG traits. However, there was no difference (*P* > 0.05) for MMW.

As shown in Table 6, the high RIG group (most efficient) had both the lowest (*P* < 0.001) feed intake (18.86 vs. 19.57 vs. 20.41 kg) and highest (*P* < 0.001) body weight gain (7.41 vs. 7.03 vs. 6.43 kg). Once again, 16-week body weight was lower (*P* < 0.001) in the high group vs. the other two RIG groups. The high RIG group also ranked best (*P* < 0.001) for FCR, RG, RFI, RIG2 and RIG3, while MMW showed no significant difference (*P* > 0.05) between the RIG groups.

Results of the phenotypic analysis of differences in feed efficiency between birds grouped based on their rank for RFI, RG, FCR, and the three RIG traits are in Table 7. Daily feed intake of the top 1% and 5% of birds was lowest when birds were ranked on RFI (0.51 and 0.60 kg), followed by RIG3 (0.55 and 0.63 kg), and was highest in the RG group (0.60 and 0.67 kg). Average daily gain was lowest at both 1% and 5% levels when ranked on RFI (0.24 kg) and highest at the 1% level when birds were ranked on RG and FCR (0.30 kg). The time required to achieve a 5 kg body weight gain for the top 1% and 5% birds was shortest (17 days) for RG, RIG2 and FCR. The feed intake to achieve a 5 kg body weight gain was lowest for the top 1% and 5% birds when ranked on FCR (9.73 and 11.04 kg). Between the residual feed efficiency traits, the top birds ranked based on RIG2 had the lowest feed intake to achieve a 5 kg body weight gain, i.e. 9.95 kg at the 1% level and 11.39 kg at the 5% level. The top birds ranked based on RFI, RG and RIG required 10.87, 10.03 and 10.07 kg, respectively, to achieve a 5 kg body weight gain at the 1% level, and 12.52, 11.48 and 11.49 kg at the 5% level.

**Table 7:** Daily feed intake (DFI), average daily gain (ADG), days to achieve 5 kg body weight gain (WG) and feed intake to achieve 5 kg body weight gain for the top 1% and 5% of 8340 tom turkeys ranked on different feed efficiency traits

**Trait**^**1**^	**DFI (kg)**	**ADG (kg)**	**Days to achieve 5 kg weight gain**^**2**^	**Feed intake (kg) to achieve 5 kg weight gain**^**3**^
**Top 1% (n = 84)**				
RFI (kg)	0.51	0.24	21	10.87
RG (kg)	0.60	0.30	17	10.03
RIG	0.56	0.28	18	10.07
RIG2	0.58	0.29	17	9.95
RIG3	0.55	0.27	19	10.20
FCR (kg/kg)	0.58	0.30	17	9.73
**Top 5% (n = 417)**				
RFI (kg)	0.60	0.24	21	12.52
RG (kg)	0.67	0.29	17	11.48
RIG	0.64	0.28	18	11.49
RIG2	0.65	0.29	17	11.39
RIG3	0.63	0.27	18	11.66
FCR (kg/kg)	0.65	0.29	17	11.04
**Bottom 5% (n = 417)**				
RFI (kg)	0.82	0.24	21	17.18
RG (kg)	0.65	0.15	34	22.36
RIG	0.76	0.20	26	19.41
RIG2	0.72	0.17	29	20.63
RIG3	0.79	0.22	23	18.37
FCR (kg/kg)	0.62	0.12	41	25.57

^1^Residual feed intake (RFI), residual body weight gain (RG), residual intake and body weight gain (RIG, RIG2 and RIG3), feed conversion ratio (FCR), average daily gain (ADG), daily feed intake (DFI); ^2^days to achieve 5 kg body weight gain (based on respective ADG); ^3^feed intake to achieve 5 kg body weight gain (days to achieve 5 kg body weight gain multiplied by DFI).

## Discussion

Estimated heritabilities for starting body weight were comparable to previous estimates in turkeys [7,11]. Similarly, estimated heritabilities for RFI, feed intake and body weight gain were also close to prior estimates in turkeys and comparable to results reported for broilers [7,12,13]. However, the heritability of RFI was lower than estimated by Aggrey et al. [14] and that of FCR (0.05) was also notably lower than previous estimates in turkeys [7]. These lower heritabilities may be due to the inclusion of all data points in this study, thereby increasing the phenotypic variance for each trait. As expected, heritabilities of all three RIG traits were similar to those estimated for the component traits (RFI and RG), since they are linear combinations of the latter. This similarity in heritabilities between RFI, RG and RIG was also previously reported in beef cattle [8,9].

The negative genetic correlation between FCR and body weight gain (−0.64) has also been observed for other meat-type poultry [12,13,15,16] and is most likely due to the inverse relationship between FCR and its component trait, i.e. body weight gain. The phenotypic and genetic correlations between FCR and RFI were lower (0.15 and 0.36) than previous estimates in turkeys and broilers [7,13,14], which is again likely due to the phenotypic variability found in FCR for the data (standard deviation of 0.96). The phenotypic correlations of zero between MMW and the residual feed efficiency traits (RFI, RG, RIG, RIG2, RIG3) were due to the inclusion of MMW in the regression model used to calculate each of these traits. For the same reason, RFI had a zero phenotypic correlation with feed intake and RG a zero correlation with body weight gain. Due to confounding factors of the RIG traits (RIG, RIG2, and RIG3) being linear combinations of RFI and RG, the phenotypic and genetic correlations of RIG were strong with both RFI and RG, and were not estimable for RIG2 and RIG3 with RFI and RG.

Potential reasons for choosing RG as a selection tool are the contrasted results observed in the phenotypic and genetic correlations between RFI and other traits of interest. If selection decisions were made purely on the basis of RFI, it is possible that slow growing birds with low feed intake rank high. The favorable genetic correlations of RG with both feed intake and body weight gain (−0.41 and 0.43), would lead selection on RG to produce faster growing birds with lower feed intake. The combination of RFI and RG into different traits (RIG, RIG2, RIG3), depending on the emphasis placed on RFI versus RG, captures the benefits of both composite traits. In contrast to RFI and RG, the RIG traits were phenotypically and genetically correlated with both feed intake and body weight gain (Table 3). The different weights on RFI and RG in the three RIG traits led to different phenotypic and genetic correlations with feed intake, with RIG2 having the weakest correlations and RIG3 the strongest correlations, with the opposite being true for correlations with body weight gain.

The use of RFI in animal breeding programs is becoming more and more prevalent across species. However, it may be more advantageous in some species than others. Comparing results from Table 4 with similarly grouped RFI animal studies in beef cattle shows a marked difference. In a study by Montanholi et al. [17], the most efficient beef bulls ranked based on RFI (low) had significantly lower DFI than the medium and high groups. This was also the case for Irish performance tested beef bulls, for which again the most efficient bulls ranked based on RFI had significantly lower DFI than the medium and high groups [9]. However, in both studies, ADG of the low RFI bulls was higher or equal than the ADG of the medium and high RFI groups. In our study on turkey, the low RFI group had significantly lower feed intake than the medium and high groups, but also had significantly lower body weight gain. This could make the use of RFI less beneficial in the turkey compared to beef cattle.

When birds were divided into groups based on RG, with the high group being the most efficient, the high group had the greatest body weight gain over the test period and a slightly lower feed intake than the medium group (Table 5). The partitioning of birds into groups based on RIG led the most efficient group (high) to have both significantly lower feed intake and significantly higher body weight gain than the medium and low groups (Table 6). This demonstrates the benefit of combining RFI and RG into a single trait. The most efficient birds based on RFI ate less but had poor body weight gain, the superior birds based on RG had the best body weight gain but poorer feed intake, and the high birds based on RIG had both excellent body weight gain and feed intake. Interestingly, when looking at the most efficient groups based on each trait, average FCR was highest for the RFI group (2.78), followed by RIG (2.57), while RG and RIG2 had the same average FCR (2.54).

As shown in Table 7, RIG2 was the closest to the ideal combination of low DFI and high ADG. While RIG2 offered the best result in this study, the weights placed on RFI and RG were not optimized; optimizing the weight could reduce feed intake to reach a 5 kg body weight gain even more. In addition, our results were based on a four-week period in the life cycle of the turkey (16–20 weeks). Thus, if the effects obtained during this period apply to the entire lifespan of the birds, cost savings due to lower consumption may be greater. Unlike RFI, RIG traits have moderate correlations (phenotypic and genetic) with body weight gain, yielding greater ADG and faster growth rates. While this can lead to improvements in feed efficiency, over-emphasizing body weight gain in a selection index may have detrimental consequences because it can have a negative impact on conformation traits such as hip and leg structure, as well as on footpad and breast skin health. Poor conformation traits can also lead to poor walking ability and other undesirable consequences [18].

Investigating the genetic and statistical parameters for traits such as RFI, RG, RIG, RIG2 and RIG3 contributes valuable knowledge to the poultry industry. However, we must also consider previous research on linearly regressed traits in animal breeding. Koch et al. [4] preferred the use of RG over RFI, both Herd et al. [19] and Arthur and Herd [1] used RFI, and Berry et al. [8] denoted the advantages of RIG. Each trait has advantages and disadvantages but all are composite traits calculated using linear regressions on the component production traits. As such, the genetic and phenotypic properties of these efficiency traits can be predicted from the genetic and phenotypic parameters of their component traits [6,20]. Adding any of the linearly regressed traits to a multiple trait selection index may prove useful but similar results may be achieved by applying the appropriate weights to their component traits (feed intake, body weight gain, metabolic mid-weight) [6].

## Conclusions

This study is the first to consider residual body weight gain and residual intake and body weight gain for poultry. Residual body weight gain was found to be moderately heritable and to have favorable phenotypic and genetic correlations with feed intake, body weight gain and FCR. Residual intake and body weight gain traits (RIG, RIG2, RIG3) were also shown to be moderately heritable, while having favorable genetic and phenotypic correlations with feed intake, body weight gain and FCR. Despite moderate to strong genetic correlations between all feed efficiency traits, none were equal to 1, indicating that, although small in some cases, there are genetic differences between these traits. Due to the strong correlations between the efficiency traits, selection on one of the feed efficiency traits will inevitably improve the others. Both RG and RIG traits have characteristics that make them appealing for inclusion in a multiple trait selection index but similar results could be attained by applying appropriate emphasis on their component traits in a selection index. Nevertheless, individual feed efficiency traits are important and perhaps the best use for RG and the three different combinations of RIG would be as benchmark traits, to compare individuals between or within flocks, and as tools to follow trends over time. Further studies on the genetic correlations between RG, RIG traits and other important traits, such as, breast meat yield, livability and walking ability are warranted.

## Competing interests

The authors declare they have no competing interests.

## Authors’ contributions

All authors were involved in the conception and design of the study. OW performed data analysis, interpretation, and wrote the manuscript. BW and SM were responsible for revisions of the manuscript drafts. All authors read and approved the final manuscript.
